# The Genetic Sphygmomanometer: an argument for routine genome-wide genotyping in the population and a new view on its use to inform clinical practice

**DOI:** 10.12688/wellcomeopenres.14870.1

**Published:** 2018-10-31

**Authors:** Nicholas John Timpson, Frank Dudbridge

**Affiliations:** 1 MRC Integrative Epidemiology Unit, University of Bristol, Bristol, UK; 2 Department of Health Sciences, University of Leicester, Leicester, UK

**Keywords:** Genetic association studies, GWAS, prediction

## Abstract

Initial genomewide association studies were exceptional owing to an ability to yield novel and reliable evidence for heritable contributions to complex disease and phenotype. However the top results alone were certainly not responsible for a wave of new predictive tools. Despite this, even studies small by contemporary standards were able to provide estimates of the relative contribution of all recorded genetic variants to outcome. Sparking efforts to quantify heritability, these results also provided the material for genomewide prediction. A fantastic growth in the performance of human genetic studies has only served to improve the potential of these complex, but potentially informative predictors. Prompted by these conditions and recent work, this letter explores the likely utility of these predictors, considers how clinical practice might be altered through their use, how to measure the efficacy of this and some of the potential ethical issues involved. Ultimately we suggest that for common genetic variation at least, the future should contain an acceptance of complexity in genetic architecture and the possibility of useful prediction even if only to shift the way we interact with clinical service providers.

Currently most genetic association studies are undertaken using common variants which tend to have small relative risks for common diseases. This prompts an approach to characterising the genetic contribution to disease through use of aggregate scores of risk alleles which can explain non-trivial proportions of variance in disease risk. Indeed the majority of common complex diseases and phenotypes are likely to be influenced by hundreds, if not thousands of common variants of small effect scattered across the genome ^1^. This means that there is information in the mass of common variants that individually are not considered to be statistically significant in a genome-wide association study. Allelic scores constructed from thousands of common variants (usually single-nucleotide polymorphisms, SNPs) across the genome can be used even without explicit knowledge of the mode of contribution of each variant, as the aggregate score uses all common genotypic contributions to explain the variance in the trait concerned ^2– 4^. Taken together, aggregate genetic risk can be one of the strongest single risk factors for a common disease, given the substantial heritability of most traits ^5^.

We should also bear in mind that most genetic association studies for complex traits are underperforming. Nearly all genetic associations are discovered through the analysis of data collected in cross-section. In most instances, prevalent cases or spot measurements are used in studies systematically hunting for correlations between phenotypes and SNPs. However, whilst it is known that genetic data is temporally stable (for the most part), the same cannot be said for phenotypic assessment. Consequently, the failure to measure outcomes adequately (i.e. in true longitudinal, or incident fashion) will at the very least compromise analytical power and cloud the inference from genetic association. Furthermore, it is increasingly clear that when longitudinal data are assessed, quite different patterns of association and variance explained can be revealed ^6, 7^. At certain points in the life course, then, the genetic risk of an individual may well be much more predictive than initially suggested by available association studies.

So if we are racing towards a world of improved genotyping scale and better studies of phenotypic association, what should we do with this information? The direct use of human genetic data in directing pharmaceutical development ^8^ and in causal inference analyses ^9^ has had a marked impact. However, if we regard genetic data as another tool of measurement in the hands of clinicians attempting to describe or predict the biological condition of an individual – the “genetic sphygmomanometer” – a natural question is: what is there to be gained from adding this particular information to the history taking process? It is well known that predicting the health trajectory of an individual is made enormously complicated by the idiosyncrasies of patients and the events of chance ^10^; so can genetic data be of any use? The answer must ultimately be yes as a more precise measurement of family history, but this does not have to be considered wholly via conventionally defined causal pathways – the detection of which suffers a natural, genetic architecture versus analytical power limited, aggregate predictive ability.

Taking the example of cancer, it is possible to generate a genome-wide aggregate score, from birth, which explains some of the risk of presenting with a malignant neoplasm later in life ^11– 13^. This is, of course, a combination of both direct pathway effects, but also relationships derived from the heritable contributions to any predicting factor – causal or otherwise. Done for each individual in a population of common ancestry to discovery studies educating the location of genetic risk, this would allow any one person to be located in a distribution of scores summarising the common genetic contribution to cancer risk. Position in this distribution is of course not a reason for immediate intervention with, say, a regime of growth factor inhibitors from the moment of full maturity, but it may offer information that could warrant something as simple as a more regular check-up regime through life. Such a non-invasive subject specific augmentation of basic clinical consultation could be the only intervention, but one which over thousands of individuals would yield life extending marginal returns at a rate greater than chance. The proposal here is that the routine incorporation of genetic data into clinicians’ hands will provide an informative addition to the standard tool kit.

Such a mechanism would be possible given two core components. The first is a centralised repository of the results of the largest, most well conducted genome-wide association studies. This would include as many disease outcomes as possible, though would only include studies of suitable rigor and with contributory information. Early versions of such repositories have already been published ^9, 14– 16^. Genetic epidemiologists have a growing collection of formidable meta-analyses that summarise effects by variant across the genome for hundreds of thousands of individuals. Made central, they could be easily accessed to provide disease specific genetic scores for any individual, which could then be used to assign that person to an augmented regime of non-invasive, lifecourse, follow-up.

Secondly, to achieve population scale benefits this approach requires all members of the population to have a comprehensive screen of common genetic variants which could be matched to the reference panel “du jour” in order to provide the material for generating individual aggregate risk scores. The actual use of these, from a consent point of view, would be the sole decision of the genotyped party (or executive) and these data could be linked to electronic health records for further clinical investigation if so required later in life or as the value of these data changes with technological advance.

A key element of this use of genetic data at population scale is the likely cost implication of personalised courses of check-ups (clinical assessment) versus the usual lifecourse clinical experience. This is a calculation which must involve a consideration of the effectiveness of altering the frequency of assessments for a person in the tails of a disease risk profile versus the cost of regular service. Factors that affect these calculations include not only the cost of assessment but also that of implementing more personalised programmes and of the resultant rates of over- and under-diagnosis of multiple conditions ^17^. However the cost of the genome-wide screening itself can be regarded as negligible once it is conducted routinely, given that the benefit is distributed across a spectrum of health outcomes, in contrast to measurement of risk markers such as lipoproteins that are specific to certain classes of disease and must be repeatedly measured over time. 

Furthermore, there is the societal or ethical cost to those located in the lower part of the risk profile. This may generate knee-jerk feelings of discrimination or injustice, however in light of our knowledge of common genetic effects of this nature (especially their interplay with the true non-genetic environment) is only as threatening as conventional diagnoses which themselves sit on a continuum of determinism. Indeed, as patients, we seem relaxed with the idea of having our own unique balance of phenotypic abnormality measured by a combination of crude instruments and clinician eye, but we remain strangely disturbed by the notion of having an additional source of information (which may at least be measured more precisely) used in the same examination.

Similarly, concerns that individuals may prefer not to know of their increased genetic risk seem to overlook how most of us live more or less easily with the knowledge of genuinely non-modifiable risk factors, age and sex. These concerns may originate from perceptions of genetic determinism occurring in rare genetic diseases, and the burden may be on genetic epidemiologists to effectively convey the probabilistic nature of genetic risk in complex disease. That said, few parents would endorse the removal of any part of a physician’s toolkit when having a child assessed or diagnosed. We should therefore in principle not feel any different about genetics when delivered appropriately. Even if it is perceived as undesirable or unethical to know genetic risk in advance of disease, once disease is established we may wish to rationalise the hand of fate by asking whether there was a genetic origin.

Can we test this approach? What guarantee is there that the formulation of differing check-up regimes will, in time, yield favourable outcomes for those subjected to them versus normal access to clinical services? The obvious step is to undertake a randomized controlled trial in a design comparable to some already ongoing in the UK and elsewhere, for example the ProtecT trial ^18^ concerning prostate cancer outcomes following randomly allocated screening. Under these conditions it would be practical to examine the impact of differential rates of clinical assessment through the lifecourse in strata assigned by aggregate genotypic scores for disease outcomes of choice. A study of this nature would not only give evidence of the value of this type of approach to realise, at population scale, the utility of the small effects of common genetic variants when combined *en masse*. It would also allow for health economic, ethical and participant experience analyses to be undertaken and further studies to be designed to bring forward genetic data as yet another part of the clinical tool kit.

This comment was first drafted in 2012 and whilst no longer potentially novel ^19, 20^, is worth blowing the dust off. In the last five years there has been an explosive development of genetic association studies, and increasing emphasis on paradigms of precision medicine. Indeed most recent initiatives in the world of genome-wide association studies have shown potential utility in prediction which may be the opening confirmation of this thesis ^21– 23^. Coupled with this is the observation that with the human genetics community continuing to pursue studies of ever increasing magnitude and power, then the ability to detect the heritable contribution to the most distal and complex of human phenotypes (including behaviour which in itself opens the door for altered environmental exposure) is increasing. Consequently the capacity for genetic studies to predict complex outcomes increases (de facto) and we are left in a position where (i) genetic prediction may not only be a flag of rare syndromes, but a reasonable guide by which one might tailor lifecourse clinical interaction, a form of “precision” medicine, (ii) we seek to identify the truly environmental factors contributing to disease as unlike measuring genetic variation, this does not have an information threshold beyond which further measurement is redundant (i.e. the challenge of tracking true non-shared environment at the individual level) and (iii) we need to challenge the inference from genetic association studies perceived to add clarity through biological/molecular insight - sampling frame, power, genetic architecture and the type of genetic study all contribute to complexities in inference ^24^. For common genetic variation and complex traits, the future should not only be causation and dissection of the wealth of new signals, but also the acceptance of complexity and the prediction of low-cost, lifecourse shifts in the way we interact with clinical service providers.

## Disclaimer

The views expressed in this article are those of the authors. Publication in Wellcome Open Research does not imply endorsement by Wellcome.

## Data availability

No data is associated with this article.
