# Supplementary material for: The Genetic Sphygmomanometer: an argument for routine genome-wide genotyping in the population and a new view on its use to inform clinical practice
Source: Wellcome Open Res. 2018 Oct 31;3:138. [Version 1] doi: 10.12688/wellcomeopenres.14870.1 (PMC6381441; doi:10.12688/wellcomeopenres.14870.1)
Supplement: Peer review history

# Peer review: Reviewer response for version 1

**DOI:** 10.21956/wellcomeopenres.16206.r34830

**Published:** 2019-02-25

**Authors:** Dorret I. Boomsma, Brandon Johnson

**Affiliations:** 1 Department of Biological Psychology, Amsterdam Public Health Research Institute, Vrije Universiteit Amsterdam, Amsterdam, The Netherlands; 2 Avera Institute for Human Genetics, Avera McKennan Hospital & University Center, Sioux Falls, SD, USA

Genomic prediction is likely to be of increasing relevance in clinical applications, prevention and to further scientific discovery. In this review, we would like to focus on three points: what might be expected when a polygenic risk score (PRS) is obtained say at birth for later outcomes in life? To answer this question we turn to twin research, in particular research with monozygotic (MZ) twins. Secondly, we want to add to the discussion on the ideal centralized repository and which members of the population should be included. Thirdly, we mention that some groups will benefit greatly from the developments in PRS research and applications as this may be their only way to learn about their genetic risks.

 More specifically to our first point of what might be expected from a PRS to tell individuals about their risk over the course of life? There probably is no better estimate for this than the concordance rates in monozygotic twin pairs: the concordance rates for disease and disorders in MZ twins gives the best limit for how predictive a PRS might be. MZ twins share (nearly) all their DNA sequence, many of the same prenatal exposures, e.g. a smoking mother, and are usually brought up in the same family. Yet, even for highly heritable traits their concordance rarely exceeds 50%. For example, for type 2 diabetes, a recent meta-analysis estimated the heritability at 72% but the concordance in MZ pairs at 2.1% (1). The heritability of schizophrenia was estimated at 79% based on data from two nationwide registries in Denmark, while the MZ twin pair concordance was 33% (2). A more comprehensive summary of MZ twin concordances is presented in reference (3) and reference (4) gives the mathematical reasoning of why this is so: If liability to disease is continuous, and disease status is diagnosed after a threshold has been passed, the  probability of discordance in MZ twins depends on the heritability of the underlying liability and on the threshold (4). Especially for rare disorders, which have a high threshold, many affected MZ twins are discordant even if the heritability is high. The reasoning that applies to an MZ twin with an affected cotwin of also becoming affected themselves, is the same reasoning that applies to an individual with a high genetic risk.

 For our second comment we would like to introduce into the discussion sample size and power versus representativeness of the proposed centralized repository. To draw accurate conclusions about a population, we do not need to include the entire population, but need to make certain that within the discovery group all individuals that reside in the population have the same chance to be part of the discovery group, e.g. through random sampling. The first issue of power receives ample attention in GWAS, the second one of representativeness currently much less.

 Lastly, will PRS replace family history? There are groups in the population for which PRS have very clear benefits. Think of individuals who were adopted, possibly from abroad, who became orphans at a young age or for other reasons do not know one or both of their biological parents. They cannot obtain their family history of disease but might learn about genetic risks through PRS.

We have read this submission. We believe that we have an appropriate level of expertise to confirm that it is of an acceptable scientific standard.

## References

[1] WillemsenGWardKJBellCGChristensenKBowdenJDalgårdCHarrisJRKaprioJLyleRMagnussonPKMatherKAOrdoňanaJRPerez-RiquelmeFPedersenNLPietiläinenKHSachdevPSBoomsmaDISpectorT: The Concordance and Heritability of Type 2 Diabetes in 34,166 Twin Pairs From International Twin Registers: The Discordant Twin (DISCOTWIN) Consortium. *Twin Res Hum Genet*.2015;18(6) : 10.1017/thg.2015.83 762-71 10.1017/thg.2015.83 26678054

[2] HilkerRHeleniusDFagerlundBSkyttheAChristensenKWergeTMNordentoftMGlenthøjB: Heritability of Schizophrenia and Schizophrenia Spectrum Based on the Nationwide Danish Twin Register. *Biol Psychiatry*.2018;83(6) : 10.1016/j.biopsych.2017.08.017 492-498 10.1016/j.biopsych.2017.08.017 28987712

[3] van DongenJSlagboomPEDraismaHHMartinNGBoomsmaDI: The continuing value of twin studies in the omics era. *Nat Rev Genet*.2012;13(9) : 10.1038/nrg3243 640-53 10.1038/nrg3243 22847273

[4] SmithC: Heritability of liability and concordance in monozygous twins. *Ann Hum Genet*.1970;34(1) :85-91 547666810.1111/j.1469-1809.1970.tb00223.x

# Peer review: Reviewer response for version 1

**DOI:** 10.21956/wellcomeopenres.16206.r34784

**Published:** 2019-02-19

**Authors:** Zoltan Kutalik

**Affiliations:** 1 Swiss Institute of Bioinformatics, Lausanne, Switzerland

The paper pinpoints the potential benefit of using genetic risk scores in clinical settings. While the outlined thoughts are individually not necessarily original, I like that these arguments are collected in a coherent piece of work. I hope that more health-economic studies will follow to assess direct health benefits of routine collection of genome-wide genetic data and providing up-to-date genetic risk to inform clinicians. Below are some suggestions that could improve the paper.

 1. Some relevant work (supporting the author’s claims) could be cited:

 Iribarren et al.(2016) ^1^

 Sanyoura et al.(2018) ^2^

 McCarthy and Mahajan (2018) ^3^

 2. Some caution would be necessary with application of such risk scores in clinical practice, some papers drawing attention to potential problems with transferring GRS from one population to another should be mentioned: e.g.

 Martin et al.(2018) ^4^. 

 Kim et al.(2018) ^5^

 Martin et al.(2017) ^6^

 3. In addition, it has to be admitted that even current studies of very large sample size tend to overestimate the importance of GRS due to uncorrected population structure, or simply GRS predicting recent migration patterns/geographic origin:

 Abdellaoui et al.(2018) ^7^

 Haworth et al.(2019) ^8^ (by the author of this work)

 Sohail et al.(2018) ^9^

 Belsky et al.(2018) ^10^

 4. The paper focused on genotyping, but could you add a few words on the benefit of sequencing?

 5. While I fully agree that routine collection of genome-wide genetic data should become the norm, improved health records on family history, health outcomes, environmental exposures, lifestyle, etc. should be equally important. For example, a part of the genetic risk score to convert to T2D is simply predicting BMI (because of the BMI->T2D causal link), hence with available BMI data, the T2D risk score should be updated accordingly.

I have read this submission. I believe that I have an appropriate level of expertise to confirm that it is of an acceptable scientific standard.

## References

[1] IribarrenCLuMJorgensonEMartínezMLluis-GanellaCSubiranaISalasEElosuaR: Clinical Utility of Multimarker Genetic Risk Scores for Prediction of Incident Coronary Heart Disease: A Cohort Study Among Over 51 Thousand Individuals of European Ancestry. *Circ Cardiovasc Genet*.2016;9(6) : 10.1161/CIRCGENETICS.116.001522 531-540 10.1161/CIRCGENETICS.116.001522 27780846

[2] SANYOURAMLETOURNEAULNAYLORRPHILIPSONLWEEDONMORAMRGREELEYS: Clinical Utility of the T1D Genetic Risk Score—Examples from the U.S. Monogenic Diabetes Registry. *Diabetes*.2018;67(Supplement 1) : 10.2337/db18-264-OR 264-OR 10.2337/db18-264-OR

[3] McCarthyMMahajanA: The value of genetic risk scores in precision medicine for diabetes. *Expert Review of Precision Medicine and Drug Development*.2018;3(5) : 10.1080/23808993.2018.1510732 279-281 10.1080/23808993.2018.1510732

[4] MartinAKanaiMKamataniYOkadaYNealeBDalyM: Current clinical use of polygenic scores will risk exacerbating health disparities. *bioRxiv*.2018; 10.1101/441261 10.1101/441261

[5] KimMPatelKTengABerensALachanceJ: Genetic disease risks can be misestimated across global populations. *Genome Biology*.2018;19(1) : 10.1186/s13059-018-1561-7 10.1186/s13059-018-1561-7 30424772PMC6234640

[6] MartinARGignouxCRWaltersRKWojcikGLNealeBMGravelSDalyMJBustamanteCDKennyEE: Human Demographic History Impacts Genetic Risk Prediction across Diverse Populations. *Am J Hum Genet*.2017;100(4) : 10.1016/j.ajhg.2017.03.004 635-649 10.1016/j.ajhg.2017.03.004 28366442PMC5384097

[7] AbdellaouiAHugh-JonesDKemperKHoltzYNivardMVeulLYengoLZietschBFraylingTWrayNYangJVerweijKVisscherP: Genetic Consequences of Social Stratification in Great Britain. *bioRxiv*.2018; 10.1101/457515 10.1101/457515 31636407

[8] HaworthSMitchellRCorbinLWadeKDuddingTBudu-AggreyACarslakeDHemaniGPaternosterLSmithGDaviesNLawsonDJ. TimpsonN: Apparent latent structure within the UK Biobank sample has implications for epidemiological analysis. *Nature Communications*.2019;10(1) : 10.1038/s41467-018-08219-1 10.1038/s41467-018-08219-1 PMC633876830659178

[9] SohailMMaierRGannaABloemendalAMartinATurchinMChiangCHirschhornJDalyMPattersonNNealeBMathiesonIReichDSunyaevS: Signals of polygenic adaptation on height have been overestimated due to uncorrected population structure in genome-wide association studies. *bioRxiv*.2018; 10.1101/355057 10.1101/355057 PMC642857130895926

[10] BelskyDDomingueBWedowRArseneaultLBoardmanJCaspiAConleyDFletcherJFreeseJHerdPMoffittTPoultonRSicinskiKWertzJHarrisK: Genetic analysis of social-class mobility in five longitudinal studies. *Proceedings of the National Academy of Sciences*.2018;115(31) : 10.1073/pnas.1801238115 E7275-E7284 10.1073/pnas.1801238115 PMC607772929987013

# Peer review: Reviewer response for version 1

**DOI:** 10.21956/wellcomeopenres.16206.r34785

**Published:** 2019-02-08

**Authors:** David J. Porteous

**Affiliations:** 1 Centre for Genomic and Experimental Medicine (CGEM), Institute of GenMolecular Medicine (IGMM), Western General Hospital, University of Edinburgh, Edinburgh, UK

I agree that genomic prediction has enormous potential, but whereas the many GWAS associations and polygenic risks scores now reported are statistically significant, the proportion of variance explained remains modest for most conditions. Moreover, there is still work to be done to answer the general question as to how cohort-based predictions translate to whole populations, or more relevantly (and not really considered in this piece) *individuals* in that population. If nothing else, the letter makes an argument for family history to be given much greater prominence in user-health service settings. We may not yet know which set of genetic variants will provide maximum predictive value, but we can certainly say that simply asking about medical histories and clinically relevant measures (BP etc) of close relatives is valuable.   

 I appreciate that this is a ‘dusting off’ of a comment first drafted in 2012, but I think it remains pertinent and I hope that my comments will be taken broadly as supportive of the proposition.

 It would be useful however to take more account of what has happened since 2012. Two obvious examples come to mind.

 The first is the release and preliminary GWAS analysis of the UK Biobank cohort (Oriol Canela-Xandri, Konrad Rawlik & Albert Tenesa (2018) ^1^) **(GeneATLAS, http://geneatlas.roslin.ed.ac.uk)

 At 500,000 strong UKB is not even 1% of the UK population, but nevertheless it should be large enough to test these predictions at a quasi-population level, for example by asking with what success do any or all polygenic risk scores predict disease incidence post-recruitment?

 The second is to acknowledge the parallel acceleration of sign-up by subscription to private sector, GWAS-based companies such as 23andMe ( www.23andme.com). The predictive validity of these over the counter tests are controversial. That, we presume, will change. Where will that lead regarding the patient-doctor relationship? Who is the guardian and who the expert of medical knowledge and wisdom.

 If GWAS is to become part of the GP tool kit then it would have to do at least three things – pass national and international standards of scrutiny for predictive validity, cross the cost/benefit boundary of clinical utility, and not add to GP consultation time. This is not to challenge the precept, but I think it would provide a useful end point to the discussion.  

 Other reviewer comments

 I endorse the comments made by fellow reviewer Nick Martin and note the author’s response.

 Minor points:

 I have a bit of a problem with the title. Anyone coming to this letter from outside scientific circles (perhaps an ALSPAC participant?) may well be put off by ‘The Genetic Sphygmomanometer’. They are more likely to read further if it were ‘The genetic blood pressure monitor’.

 Some of the text is a bit convoluted.

 ‘the detection of which suffers a natural, genetic architecture versus analytical power limited, aggregate predictive ability.’ Is convoluted and opaque. Please rephrase [sometimes two sentences are better than one]

 ‘Done for each individual in a population of common ancestry to discovery studies educating the location of genetic risk,’ is convoluted. Please rephrase. My suggestion: ‘If done for each individual in a population of common ancestry to define the genetic risk loci,’

 ‘for those subjected to them versus normal access’ sounds like a punishment. Perhaps ‘for those offered them versus normal access’?

 ‘an explosive development of’  ‘an explosion of’ and add current number with date.

I have read this submission. I believe that I have an appropriate level of expertise to confirm that it is of an acceptable scientific standard, however I have significant reservations, as outlined above.

## References

[1] Canela-XandriORawlikKTenesaA: An atlas of genetic associations in UK Biobank. *Nat Genet*.2018;50(11) : 10.1038/s41588-018-0248-z 1593-1599 10.1038/s41588-018-0248-z 30349118PMC6707814

# Peer review: Reviewer response for version 1

**DOI:** 10.21956/wellcomeopenres.16206.r34148

**Published:** 2018-11-05

**Authors:** Nicholas G. Martin

**Affiliations:** 1 Queensland Brain Institute, University of Queensland, Brisbane, Qld, Australia

3rd para, [beg So if we..] last sentence is so convoluted as to be incomprehensible; please rephrase.

 6th para [beg Secondly..] Why does it need to be compulsory ["requires all members"] ? Why cant it be voluntary and incremental? Of course it would be best if 100% did it, but there's utility in less than perfect, and much more acceptable to the populace.

 7th para [beg A key..]; authors might consider the influence of neurotic/conscientious vs. lackadaisical/thick in uptake and use; these are biases that can be quantified from known PRS.

 8th para [beg Furthermore..] Not clear which is lower and upper tail; is lower = higher risk ? If lower = lower risk then these people will feel relief, surely.

 9th para [beg Similarly]: "concerns that indivs.." Why should there be concern? It's an individual choice and people can pick and chose what they want to know. For example, they may wish to know their PRS for glaucoma [actionable] but not for Alzheimer's [not].

 10th para [beg  Can we test] what do they speculate on is already happening - lots at ASHG this year ^1^.

 Last paragraph, last sentence; couldn't agree more!

I have read this submission. I believe that I have an appropriate level of expertise to confirm that it is of an acceptable scientific standard.

## References

[1] BrownSNJouniHMarroushTSKulloIJ: Effect of Disclosing Genetic Risk for Coronary Heart Disease on Information Seeking and Sharing: The MI-GENES Study (Myocardial Infarction Genes). *Circ Cardiovasc Genet*.2017;10(4) : 10.1161/CIRCGENETICS.116.001613 10.1161/CIRCGENETICS.116.001613 28779015PMC5547821

# Response

**Published:** 2018-12-10

**Authors:** Nic Timpson

**Affiliations:** University of Bristol, UK

Response to initial review (NM)

3rd para, [beg So if we..] last sentence is so convoluted as to be incomprehensible; please rephrase.

- This has been attended to and will be included in the next posted version.

6th para [beg Secondly..] Why does it need to be compulsory ["requires all members"] ? Why cant it be voluntary and incremental? Of course it would be best if 100% did it, but there's utility in less than perfect, and much more acceptable to the populace.

- This is absolutely right of course. Our phrasing:

“this approach requires all members of the population to have a comprehensive screen of common genetic variants”

… was really to assert that the approach here would be hypothetically a population based effort which would provide best utility for understanding the complex contributions to complex health outcomes in all. Indeed this is far from deterministic and one would have to operate efficient and population scale changes in health care interaction to see on average benefits. We agree with the point that there is an ideal scenario that would have everyone participating, but in reality an optional scheme is more acceptable and would still be useful.

7th para [beg A key..]; authors might consider the influence of neurotic/conscientious vs. lackadaisical/thick in uptake and use; these are biases that can be quantified from known PRS.

- This is a sensible and real detail which would have to be accounted for in any real-world development of this type of approach to incorporating genetic information into health care practice in the proposed way. Key to this, however, is the step-change notion that genetics (even of complex traits) is not to be treated in a manner different to other forms of partial diagnosis and medical examination. These features also suffer the same issues, though are not abandoned as a result.

8th para [beg Furthermore..] Not clear which is lower and upper tail; is lower = higher risk ? If lower = lower risk then these people will feel relief, surely.

- Either way there is of course the interesting position that those with low predicted risk will (a) feel potential relief, but (b) will have to understand their relative risk position and that this is a predicted average. For clarification, being “located in the lower part of the risk profile” does mean at lower predicted risk and could potentially elicit feelings of discrimination in that other people are getting more attention when they themselves remain at risk. This is a central tension in that the probabilistic assessment of risk will not satisfy individual feelings of need for health care – nor should it preclude access. There is a need to discuss the potential impact of even small changes of behaviour with relation to interaction with healthcare in light of this – though it seems less likely that those at (on average) lower risk would chose to alter their use of healthcare provision.

9th para [beg Similarly]: "concerns that indivs.." Why should there be concern? It's an individual choice and people can pick and choose what they want to know. For example, they may wish to know their PRS for glaucoma [actionable] but not for Alzheimer's [not].

- This paragraph was not as clear as it could have been and will be updated in the next posted version. The concerns mentioned refer to both those of the clinician and the patient/health member of the public and you are right – there should be no concern that some chose not to know of risk.Individuals clearly should have the choice whether to be informed of their genetic risk, but that choice should itself be informed not only by the potential impact to health individually, but also the potential benefit to the population more broadly. 

10th para [beg  Can we test] what do they speculate on is already happening - lots at ASHG this year ^1^.

Last paragraph, last sentence; couldn't agree more!

- It is not clear that there are explicit trials of this type of activity currently, but this could be undertaken. Further to this – we are delighted that the reviewer agrees with our last point.

** of note – all manuscript changes will be made/updated on the receipt of 3 reviewer contributions.
